# Targeted Knockdown of the Kinetochore Protein D40/Knl-1 Inhibits Human Cancer in a p53 Status-Independent Manner

**DOI:** 10.1038/srep13676

**Published:** 2015-09-08

**Authors:** Yuri N. Urata, Fumitaka Takeshita, Hiroki Tanaka, Takahiro Ochiya, Masato Takimoto

**Affiliations:** 1 Division of Cancer Gene Regulation, Institute for Genetic Medicine, Hokkaido University, Sapporo, Hokkaido, Japan; 2 Division of Molecular and Cellular Medicine, National Cancer Center Research Institute, Tokyo, Japan; 3 Department of Functional Analysis, National Cancer Center Research Institute, Tokyo, Japan

## Abstract

The D40 gene encodes a kinetochore protein that plays an essential role in kinetochore formation during mitosis. Short inhibitory RNA against D40, D40 siRNA, has been shown to deplete the D40 protein in the human cancer cell line HeLa, which harbors wild-type p53, and this activity was followed by the significant inhibition of cell growth and induction of apoptotic cell death. The p53-null cancer cell line, PC-3M-luc, is also sensitive to the significant growth inhibition and cell death induced by D40 siRNA. The growth of PC-3M-luc tumors transplanted into nude mice was inhibited by the systemic administration of D40 siRNA and the atelocollagen complex. Furthermore, D40 siRNA significantly inhibited growth and induced apoptotic cell death in a cell line with a gain-of-function (GOF) mutation in p53, MDA-MB231-luc, and also inhibited the growth of tumors transplanted into mice when administered as a D40 siRNA/atelocollagen complex. These results indicated that D40 siRNA induced apoptotic cell death in human cancer cell lines, and inhibited their growth *in vitro* and *in vivo* regardless of p53 status. Therefore, D40 siRNA is a potential candidate anti-cancer reagent.

We previously described the cloning of the human gene D40 on chromosome 15^1^, and we characterized D40 as a member of the cancer/testis gene family that is predominantly expressed in the testis and widely expressed in various cultured human cancer cell lines and primary tumors of different origins^2^,^3^. Hayette *et al.* reported a gene that was mutually translocated with the MLL (mixed lineage leukemia) gene in acute myeloid leukemia^4^. The gene on human chromosome 15, AF15q14, was identical to the D40 gene. D40 expression has been correlated with the clinicopathological characteristics of primary lung tumors, such as the degree of differentiation and the smoking habits of patients^3^. In the testis, marked expression of the D40 protein was observed in spermatocytes and the pre-acrosomes of spermatids^5^. Subsequent studies revealed that a kinetochore protein in the mitotic machinery, Knl-1/Blinkin, was identical to D40. Knl-1/Blinkin binds not only to Mis12 and Ndc80 complexes, which comprise the KMN network, but also to several other proteins including tubulin, the spindle assembly checkpoint (SAC) proteins Bub1 and BubR1, and Protein Phosphatase 1. By binding to these proteins, Knl-1/Blinkin plays critical roles in kinetochore formation, connecting chromosomes and spindles and regulating SAC^6^,^7^,^8^,^9^,^10^,^11^.

Primary microcephaly (MCPH) is a very rare congenital neuro-developmental disorder with autosomal recessive traits^12^,^13^. Patients with MCPH have a small brain and a reduced head circumference, which are associated with mild to moderate mental retardation, but these patients do not have additional neurological or somatic diseases. MCPH is genetically heterogeneous with several responsible chromosomal loci. A locus of MCPH was recently mapped to chromosome 15^13^, and the responsible gene was identified as CASC5, which is also identical to D40^14^. Given that rapid cell division is a necessary characteristic of a developing brain, these observations provide further evidence that D40 plays an important role in cell division *in vivo*.

As a tumor suppressor, the p53 protein plays essential roles in various cell biological processes, such as cell cycle regulation, DNA damage repair and stress responses^15^,^16^,^17^. Accordingly, mutations in the p53 gene in tumor cells contribute to the development of tumors and malignant phenotypes. Functionally, the p53 status of tumor cells has been classified into three types: wild-type, loss-of-function (LOF) mutants, which include recessive and null types, and gain-of-function (GOF) mutants. Of these, GOF mutants confer the most malignant phenotypes on tumors, including resistance to chemotherapy^18^,^19^. Although recent developments in molecular targeted drugs appear to be encouraging, most patients eventually develop resistance to these drugs. Recent studies have shown that wild-type p53 increases the sensitivity of tumor cells to molecular targeted drugs, and p53 mutation conferred resistance to these drugs^20^,^21^,^22^,^23^.

In the present study, we showed that the depletion of the D40 protein by short inhibitory RNA (siRNA) significantly inhibited the growth and induced apoptotic cell death in cancer cell lines in a p53 status-independent manner *in vitro* and also markedly impeded the growth of *in vivo* transplanted tumors. We propose that D40 siRNA may be a potential candidate for cancer therapy.

As described above and previously^1^,^3^,^4^,^6^,^9^, D40 has several aliases; AF15q14, KIAA1570, CASC5, Blinkin, and Knl-1. In the present study, we used the nomenclature D40.

## Results

**Figure 1:**
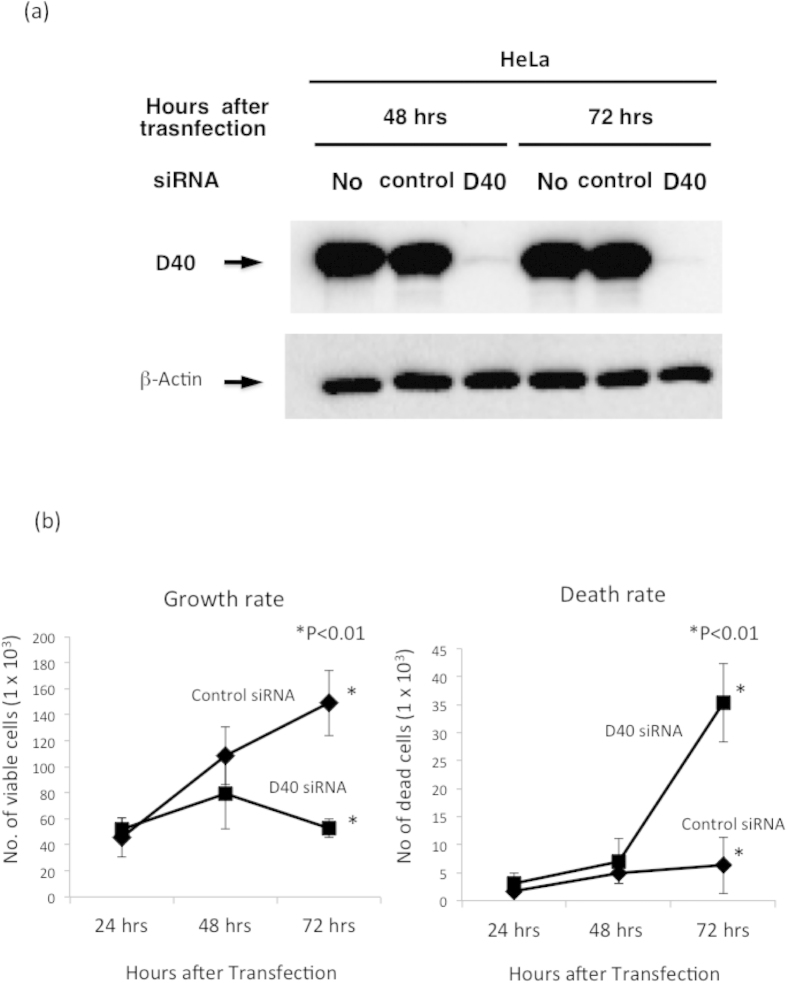
Growth Inhibition and Induction of Apoptotic Cell death by D40 siRNA in the wild-type p53 cancer cell line. (**a**) Depletion of D40 protein by D40 siRNA. The human cancer cell line with wild-type p53, HeLa, was transfected with D40 or control siRNA. The transfected HeLa cells were harvested at the indicated time points after transfection, and cell lysates were prepared with TNE buffer. The gels for D40 and β-actin were run under the same experimental conditions, and the proteins were transferred to a filter membrane. The membrane was cut and divided into two parts. One was probed with the anti-D40 antibody and the other was probed with the anti-β-actin antibody. Significant depletion of D40 protein was observed in D40 siRNA-transfected HeLa cells. (**b**) Growth and Death Rate Curves of HeLa cells transfected with D40 siRNA. HeLa cells were transfected with D40 or control siRNA and harvested at the indicated times after transfection. The number of viable cells and dead cells was significantly lower and higher, respectively, in D40 siRNA-transfected HeLa cells than in control siRNA-transfected cells 72 hrs after transfection.

RNA interference targeting D40 was used to determine whether inhibiting D40 protein expression affected the growth of human cancer cell lines. Three different D40 mRNA sequences that were 21 bases in length were selected to synthesize short inhibitory RNA (siRNA). A mixture of the siRNAs (D40 siRNA) was transfected into the wild-type p53 cell line HeLa. D40 siRNA, but not control siRNA, significantly reduced D40 protein expression at the indicated hours after transfection (Fig. 1a). As the specific depletion of the D40 protein was induced by D40 siRNA, the effects of D40 siRNA on the growth of the HeLa cell line were assessed. The growth of HeLa cells was significantly inhibited by D40 siRNA, but not by control siRNA (Fig. 1b Left). The proportion of dead cells was higher in D40 siRNA-transfected HeLa cells than in control HeLa cells (Fig. 1b Right). Each of the three D40 siRNAs also significantly depleted D40 protein expression and inhibited the growth of HeLa cells (Supplementary Figures 1 and 2). The mixture of three different D40 siRNAs was labelled as D40 siRNA Trio in Supplementary Figures 1 and 2. However, this trio was described as D40 siRNA in all of the other parts of the manuscript and figures.

**Figure 2:**
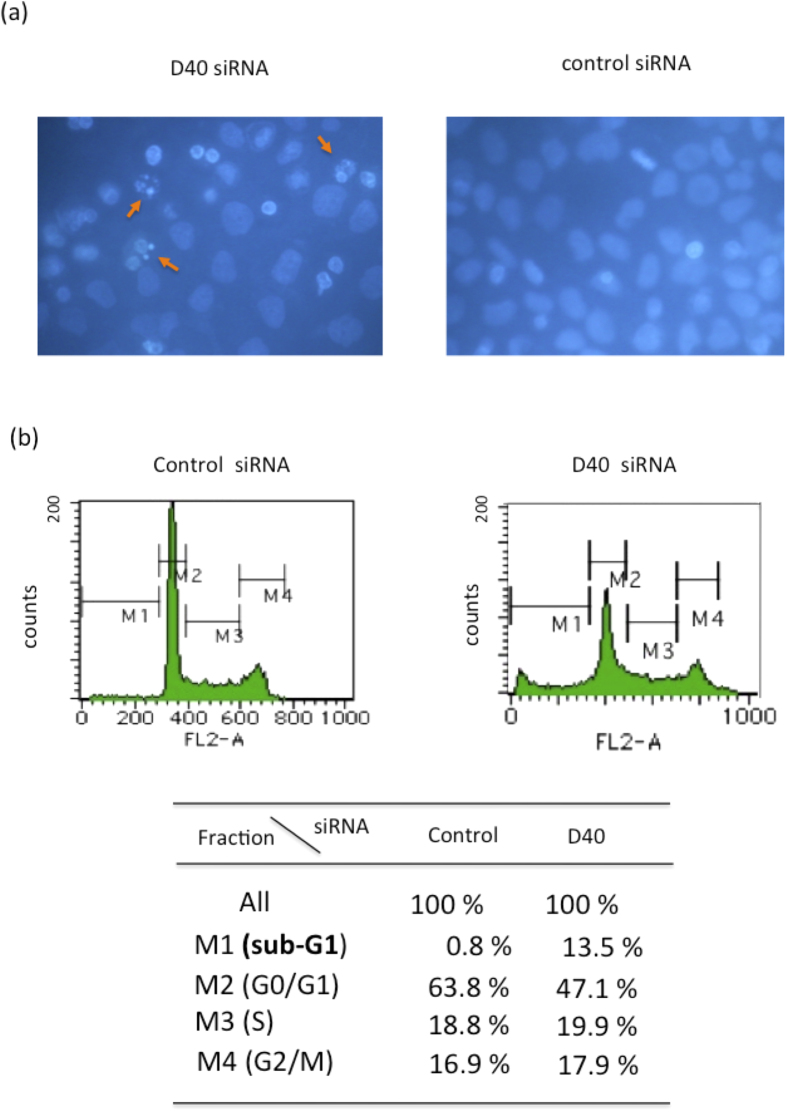
Induction of Apoptotic Cell death by D40 siRNA in the wild-type p53 cancer cell line. (**a**) Nuclear chromatin condensation and fragmentation induced by D40 siRNA in HeLa cells. HeLa cells were transfected with D40 siRNA (left) or control siRNA (right), and were stained with Hoechst 33342 48 hrs after transfection. Cells with fragmented chromatin were regarded as apoptotic. Arrows in the left figure indicate the apoptotic D40 siRNA-transfected cells. (**b**) Increase in the sub-G1 fraction of HeLa cells transfected with D40 siRNA. DNA content analyses were performed as described in the Materials and Methods section. The upper figures show histograms of the DNA contents of cells 48 hrs after transfection, while the lower table shows the percentage of the cell population in each fraction. A significant increase in the sub-G1 fraction was detected in the D40 siRNA-transfected HeLa cells but not in control siRNA-transfected cells.

The nuclear staining of siRNA-transfected HeLa cells was performed using Hoechst 33342, and the results obtained revealed that a greater population of the cells with chromatin condensation and fragmentation, which are features of apoptotic cells, was observed in the D40 siRNA-transfected cells compared with control cells (Fig. 2a). To confirm cell death in D40 siRNA-transfected cells, DNA content analyses of the transfected cells were performed using a flow cytometer. The population of cells with a DNA content less than 2n, the sub-G1 population in the M1 fraction, was significantly higher in D40 siRNA-transfected cells than in cells transfected with control siRNA, as shown in Fig. 2b. As the sub-G1 population is characteristic of apoptosis, we concluded that D40 siRNA induced apoptotic cell death in HeLa cells by apoptosis.

p53 plays a crucial role in apoptosis, and mutations in the p53 gene are known to cause the development of malignant phenotypes in tumors, such as resistance to therapies. Mutations in the p53 gene have been reported in approximately 50% of all primary human cancers; therefore, it is very important and necessary to develop anti-cancer therapeutic strategies that are effective against tumors that bear mutations in the p53 gene and became resistant to therapies.

**Figure 3:**
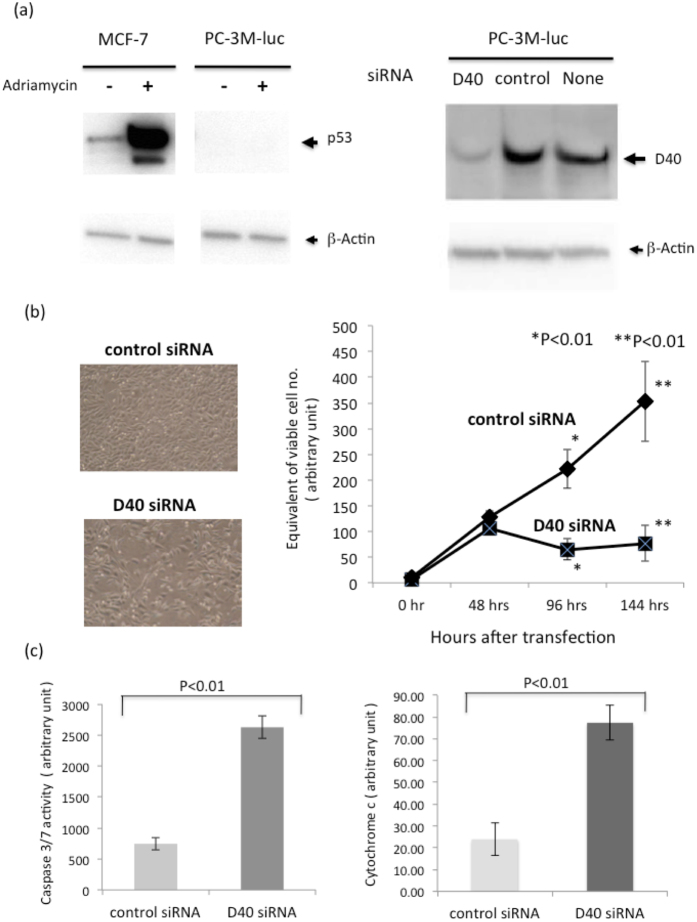
D40 siRNA induces Growth Inhibition and Apoptotic Cell death in the p53-null cancer cell line. Confirmation of the p53-null state of the PC-3M-luc cell line and depletion of the D40 protein by D40 siRNA. Left: The human p53-null cancer cell line PC-3M-luc and human wild-type p53 cancer cell line MCF-7 were treated with adriamycin at a final concentration of 1.0 μg/ml for 17 hrs. Cell lysate preparation and WB analyses were performed as described above. The gels for p53 and β-actin were run under the same experimental conditions, and then the proteins were transferred to a filter membrane. The membrane was cut and divided into two parts. One was probed with the anti-p53 antibody, and the other was probed with the anti-β-actin antibody. p53 protein was absent in PC-3M-luc cells, but p53 protein expression was highly induced in MCF-7 cells. Right: PC-3M-luc cells were transfected with D40 or control siRNA and harvested 60 hrs after transfection for WB analyses. The gels for D40 and β-actin were run under the same experimental conditions, and then the proteins were transferred to a filter membrane. The membrane was cut and divided into two parts. One was probed with the anti-D40 antibody, and the other was probed with the anti-β-actin antibody. (**b**) Growth Inhibition of the PC-3M-luc cell line by D40 siRNA. Left: Microphotographs of the PC-3M-luc cell line transfected with control RNA (upper) or D40 siRNA (lower) 60 hrs after transfection. Right: Growth curves of the transfected PC-3M-luc cell lines. Cell counting kit-8 was used to determine viable cell numbers. The number of viable cells 96 hrs and 144 hrs after transfection was significantly lower in the D40 siRNA-transfected PC-3M-luc cell line than in the respective control cell lines. (**c**) D40 siRNA induced the activation of caspase 3/7 and increased cytoplasmic cytochrome c levels in the PC-3M-luc cell line. The PC-3M-luc cell line was transfected with siRNAs as described. Left: Caspase 3/7 activities in the transfected PC-3M-luc cells were determined 96 hrs after transfection. Right: The amount of cytoplasmic cytochrome c was quantitatively determined 70 hrs after transfection.

We first assessed the growth inhibitory effects of D40 siRNA on the p53-null cell line, PC-3M-luc, which represents a loss-of-function (LOF) p53 mutants cell line^24^. To confirm that this cell line did not express the p53 protein, Western blot analysis was performed on the lysates of cells that were treated with adriamycin, a DNA-damaging reagent. While the p53 protein was highly induced the adriamycin treatment in the wild-type p53 cell line MCF-7, it was undetectable in the PC-3M-luc cell line, even under the same treatment conditions (Fig. 3a Left). We subsequently referred to the PC-3M-luc cell line used in this study as p53-null. We next determined whether D40 siRNA could reduce D40 protein expression in this cell line. The results demonstrated that D40 protein expression in the PC-3M-luc cell line was sensitive to D40 siRNA (Fig. 3a Right). Therefore, the effects of D40 siRNA on the growth of this cell line were examined. The results showed that growth was significantly inhibited in the cells transfected with D40 siRNA, as shown in the microphotographs (Fig. 3b Left) and growth curves (Fig. 3b Right).

We next investigated whether the D40 siRNA-mediated growth inhibition in the p53-null cell line occurred via apoptosis and attempted to identify which apoptotic signal pathway led to PC-3M-luc cell death in response to D40 siRNA. Two major apoptotic pathways have been detected in mammalian cells—an intrinsic pathway mediated by mitochondria and an extrinsic pathway involving a cell surface receptor. The mitochondria-mediated intrinsic pathway is known to respond to a wide range of death stimuli, and this pathway has been subdivided into at least two sub-pathways. The first intrinsic sub-pathway is mediated by cytochrome c, which subsequently activates Apaf1, caspase 9, and caspase 3/7. The second intrinsic sub-pathway is mediated by Apoptosis-Inducing Factor (AIF), which activates Endonuclease G (Endo G), a caspase-independent DNase^25^,^26^. To examine the apoptotic pathway caused by D40 siRNA, we examined whether caspase 3/7 was activated in D40 siRNA-transfected cells compared with control siRNA-transfected cells. The results revealed that caspase 3/7 activity was significantly higher in the former than in the latter, which excluded the involvement of the second sub-pathway (Fig. 3c Left). We also observed a higher quantity of cytoplasmic cytochrome c in PC-3M-luc cells transfected with D40 siRNA than in those transfected with control siRNA (Fig. 3c Right). These results suggested that the cell death induced by D40 siRNA occurred through the first intrinsic sub-pathway, which is mediated by mitochondria, cytochrome c and caspase 3/7.

**Figure 4:**
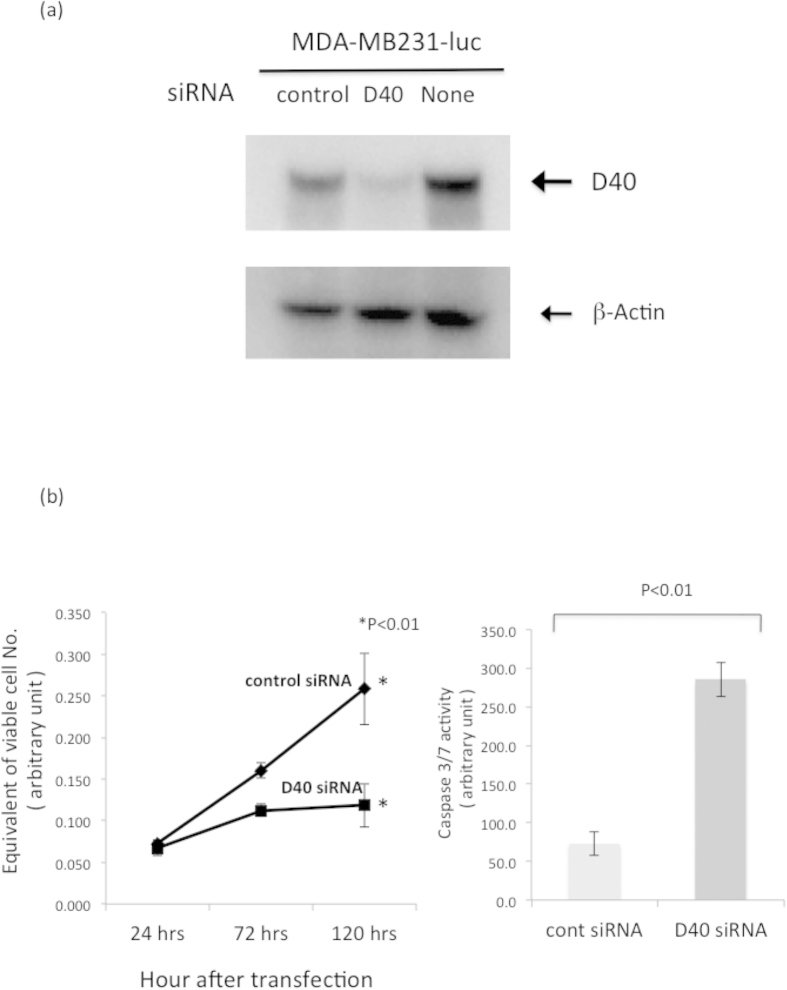
Gain-of-Function (GOF) mutant p53 cell line was susceptible to Growth Inhibition and Apoptotic Cell death by D40 siRNA. (**a**) The GOF mutant p53 cell line MDA-MB231-luc was susceptible to depletion of D40 protein by D40 siRNA. MDA-MB231-luc cells were transfected with D40 or control siRNA, and WB analyses of D40 protein expression were performed 48 hrs after transfection. The gels for D40 and β-actin were run under the same experimental conditions, and then the proteins were transferred to a filter membrane. The membrane was cut and divided into two parts. One was probed with the anti-D40 antibody and the other was probed with the anti-β-actin antibody. (**b**) D40 siRNA induced Growth Inhibition and Caspase 3/7 activation in the MDA-MB231-luc cell line. Left: Growth curves of the MDA-MB231-Luc cell line. The transfection of siRNAs and the determination of the viable cell number were performed as described in Fig. 2B. Right: Ninety-six hours after transfection, the cells were harvested and caspase 3/7 activities were determined.

We next determined whether D40 siRNA inhibited the growth of the GOF p53 mutant cell line MDA-MB231-luc, which exhibits malignant phenotypes such as a high potentials for invasiveness and metastasis as well as resistance to therapeutics. As such, it was important to assess the sensitivity of this cell line to D40 siRNA. The results revealed that D40 siRNA could significantly reduce D40 protein expression (Fig. 4a). The growth of cells transfected with D40 siRNA was also inhibited compared with that of cells transfected with control siRNA (Fig. 4b Left). Furthermore, caspase 3/7 activity was higher in cells transfected with D40 siRNA than in those transfected with control siRNA (Fig. 4b Right). These results indicated that D40 siRNA effectively induced apoptosis in tumors with a GOF mutation in p53.

**Figure 5:**
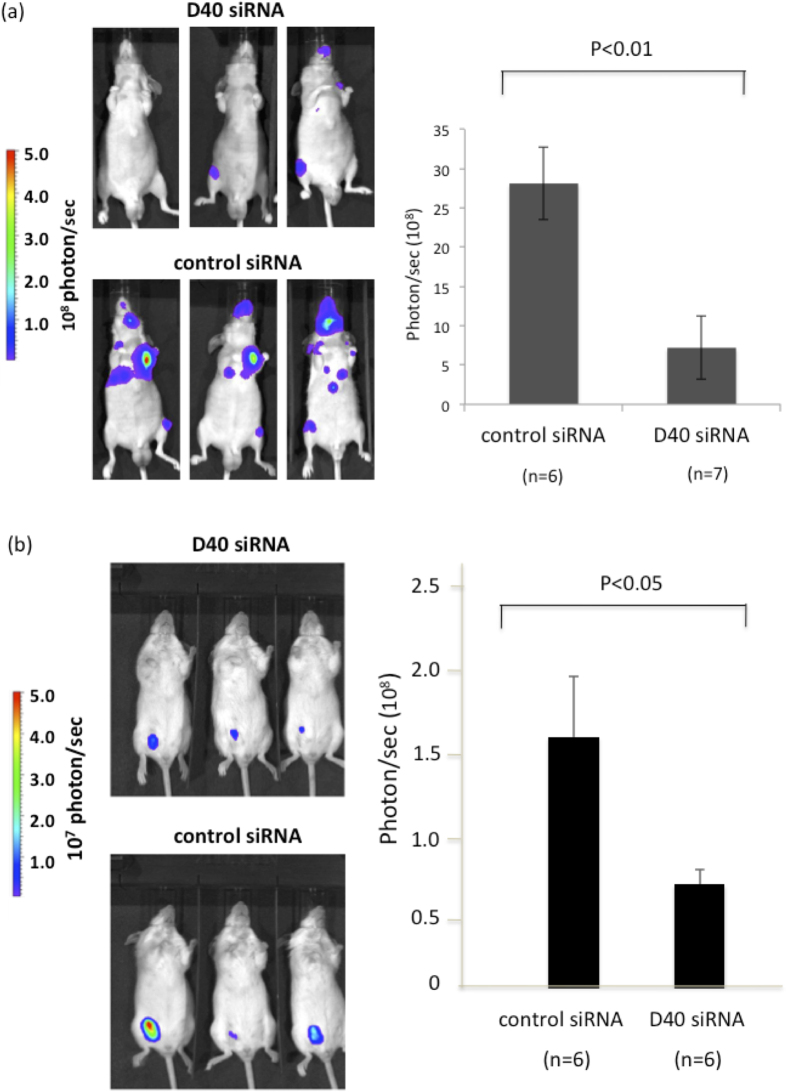
*In vivo* Growth Inhibition of p53 mutant cancer cell lines by D40 siRNA. (**a**) D40 siRNA inhibited the metastatic growth of the PC-3M-luc p53-null cell line after transplantation into nude mice. Two million PC-3M-luc cells were transplanted into the left cardiac ventricle of nude mice. The siRNA and atelocollagen complex was injected into the tail veins of mice 4, 7, 13, and 16 days after transplantation. On day 29, the bioluminescence from the tumors was quantitatively detected. Left: Representative bioluminescent images of mice treated with the D40 siRNA/atelocollagen complex (upper) or the control (lower). Right: Statistical analysis of bioluminescent signals from the tumors in mice treated with the D40 siRNA/atelocollagen complex versus the control. Data represent the mean ± sd. (**b**) D40 siRNA inhibited the growth of the MDA-MB231-luc GOF mutant of p53 cell line after transplantation into scid mice. One million MDA-MB231-luc cells were transplanted into the mammary glands of scid mice. The siRNA and atelocollagen complex was injected into the tumors on mice 4, 6, 8, 11, 13, 15, and 18 days after transplantation. On day 18, the bioluminescence from the tumors was quantitatively detected. Left: Representative bioluminescent images of mice treated with D40 siRNA (upper) or control siRNA (lower). Right: Statistical analysis of the bioluminescent signals from the tumors in mice treated with the D40 siRNA/atelocollagen complex versus the control. Data represent the mean ± se.

In subsequent experiments, we determined whether D40 siRNA could inhibit the growth of tumors transplanted in experimental animals. The p53-null PC-3M-luc cells were inoculated into the left cardiac ventricles of nude mice, and the D40 siRNA/atelocollagen complex, in which atelocollagen was used as a carrier for the siRNA, was then injected intravenously to examine the effects of D40 siRNA on the metastatic growth of tumors in nude mice^27^,^28^. As PC-3M-luc cells were engineered to constitutively express luciferase, the growth of tumors in nude mice was monitored using an extracorporeal device after the injection of a substrate for luciferase. Representative extracorporeal bioluminescent images of tumors in mice are shown in Fig. 5a Left. The bioluminescent signals observed in mice treated with the D40 siRNA/atelocollagen complex (upper) were significantly weaker than those in control mice (lower). Statistical analysis of the quantified bioluminescent signals from mice revealed that metastatic growth of the PC-3M-luc cell line was significantly inhibited by the D40 siRNA/atelocollagen complex compared with control (Fig. 5a Right).

We next examined whether the growth of GOF p53 mutant cells MDA-MB231-luc cells transplanted into mice could be inhibited by the administration of the D40 siRNA/atelocollagen complex. MDA-MB231-luc cells that also constitutively expressed luciferase were transplanted into the mammary glands of scid mice, and the D40 siRNA/atelocollagen complexes were then administered into the tumors. The growth of the transplanted tumors in scid mice was examined extracorporeally, and representative bioluminescent images of tumors in mice are shown in Fig. 5b Left. Bioluminescent signals from mice treated with D40 siRNA/atelocollagen complexes (upper) were significantly weaker than those from control mice (lower). Statistical analysis of the quantified signals from mice revealed that the growth of MDA-MB231-luc cells was significantly less in the presence of the D40 siRNA/atelocollagen complexes compared with the control (Fig. 5b Right).

## Discussion

In the present study, we demonstrated that D40 siRNA, a D40-specific short inhibitory RNA, markedly inhibited the growth of human cancer cell lines both *in vitro* and *in vivo* by inducing apoptosis in a p53 status-independent manner. Although previous papers described the abnormal mitosis in cancer cells treated with D40/Knl-1 siRNA^9^,^29^, we are the first to demonstrate that D40 siRNA induced apoptosis in and inhibited the growth of cancer cell lines, not only *in vitro*, but also in animal experiments. This was also found to occur regardless of the p53 status of the cancer cell lines.

The animal experiment in which PC-3M-luc cells were transplanted with intracardiac injection is recognized as an established system for evaluating bone metastasis in conjunction with studies that include detailed histological examinations, such as HE staining^27^,^30^,^31^. The histological data on bone metastasis in our previous experiment are shown as a reference in Supplementary Figure 11. Therefore, it is reasonable to consider that the bioluminescent signals from the mice in this study are derived mainly from bone metastases. Although the experimental system we used in this study is a model of bone metastasis, it can be utilized not only for evaluating bone metastasis but also for detecting micro tumors in the mice, which are difficult to detect by histological analysis, and for evaluating the growth of the tumors.

Regarding the apoptotic mechanism, Kitagawa *et al.* demonstrated that cell death occurred in mitosis through the second intrinsic sub-pathway involving AIF and Endo G, and this was independent of caspase and p53, but dependent on p73 under the conditions in which the mitotic checkpoint protein Bub1 was partially depleted. They termed this cell death caspase-independent mitotic death (CIMD)^32^. The cell death induced by D40 siRNA was shown to occur independently of p53, and D40 was reported to bind to Bub1^9^; therefore, we examined whether cell death in the p53-null PC-3M-luc cells was caused by CIMD or through the first intrinsic caspase-dependent sub-pathway. The results showed that cell death in PC-3M-luc cells as well as the GOF p53 mutant cell line, MDA-MB231-luc, was mediated through the first intrinsic caspase-dependent sub-pathway (Figs 3c and 4b Right). We also examined the D40 siRNA-induced apoptotic pathway in HeLa cells, which harbor wild-type p53, and the results revealed that cell death in this cell line occurred through the first intrinsic sub-pathway and was mediated by caspase 3/7 and cytochrome c (Supplementary Figure 3, 4). It was reported that CIMD was dependent on certain conditions within tumors, such as the partial depletion of Bub1^33^; therefore, the result that D40 siRNA-mediated cell death was executed by a more common apoptotic pathway than CIMD is advantageous for the application of D40 siRNA as an anti-cancer therapy.

Although it has been more than 20 years since p53 was established as a tumor suppressor^17^, the roles of p53 have continued to increase, mainly involving its functions as a transcription factor. Early studies on wild-type p53 demonstrated that this tumor suppressor plays essential roles in DNA damage and repair responses, cell cycle regulation, the induction of apoptosis, sensitivity to chemo- and radiotherapy, and the inhibition of angiogenesis^15^,^16^,^17^. Recent studies have shown that p53 is crucially involved in regulating cell metabolism, the production of reactive oxygen species (ROS), senescence, autophagy, and stem cell fate, including in iPS cells^34^,^35^,^36^,^37^. p53 was more recently shown to regulate miRNA^38^. Furthermore, a recent study demonstrated that p53 activates a mitochondrial protease that cleaves β-actin, thereby inhibiting the invasion of ras-transformed cells^39^.

Based on the broad spectrum of these functions, the LOF and GOF p53 mutations contribute to the transformation of cells and confer the utmost malignant phenotypes on tumor cells, such as a high growth rate, extraordinary invasive and metastatic abilities, and obstinate resistance to radiation and chemotherapeutic agents^18^. Recent studies have elucidated the molecular mechanisms responsible for metastasis promoted by mutant p53^19^,^40^,^41^,^42^,^43^,^44^,^45^,^46^, which interacts with and activates or down-regulates a wide variety of cellular proteins.

The treatment of advanced malignant cancers with molecular targeted drugs that inhibit pro-metastatic molecules is anticipated. However, two or more drugs may be needed to treat a patient because metastasis is a multi-step phenomenon. Different drugs may also be necessary for each tumor lineage. By contrast, D40 siRNA has several advantages in the treatment of advanced cancers. First, D40 is a kinetochore protein that plays a key role in mitotic cell division, which is an essential step in cell growth for all cancers. As advanced cancers divide frequently, the depletion of D40 protein would be considerably effective at inhibiting the growth of tumors by directly inhibiting the mitotic machinery and subsequently inducing apoptotic cell death. Second, as we showed in the results of this study, D40 siRNA inhibits tumors with metastatic potential and with the GOF mutant of p53, and it is likely that tumors with a high malignant phenotype are sensitive to D40 siRNA. Third, as the inhibitory effects of D40 siRNA are p53-status independent and about one-half of primary cancers harbor a p53 mutation, it is expected that several clinical cancers would respond to the growth inhibitory effect of D40 siRNA.

Of particular importance regarding the treatment of advanced cancer is that p53 has been shown to play key roles in cancers therapy, even with molecular targeted drugs. Functional p53 was shown to play a critical role in the therapeutic effects of the synthetic Epidermal Growth Factor Receptor (EGFR) inhibitor gefitinib by enhancing the apoptotic pathway in non-small cell lung cancer^20^,^21^. Another study reported that p53, together with PTEN, contributed to the inhibition of signaling pathways downstream of EGFR by cetuximab, a monoclonal anti-EGFR, in prostate cancer^22^. Huang *et al.* demonstrated that p53 was markedly down-regulated in the EGFR inhibitor-resistant clones of a human lung cancer cell line and that silencing p53 in parental cells reduced their sensitivity to cetuximab^23^. The introduction of wild-type p53 into the cetuximab-resistant clones was sufficient to restore their sensitivity to the EGFR inhibitor. The study indicated that the p53 mutation conferred tumors resistance, even to molecular targeted therapies, which are at the forefront of current cancer therapies. Because D40 siRNA can inhibit tumor growth and induce apoptosis regardless of the p53 status, D40 siRNA may be able to inhibit the growth of tumors that have become resistant to molecular targeted drugs, such as EGFR inhibitors.

In summary, we demonstrated that the targeted knockdown of kinetochore protein D40 inhibits human cancers *in vitro* and *in vivo* in a p53 status-independent manner. p53 is the most frequently mutated cancer-related gene; it exhibits not only LOF but also GOF mutations, thereby conferring highly malignant phenotypes on tumors^19^,^40^. Therefore, it is essential to develop anti-cancer agents that are effective in tumors that bear p53 gene mutations and that have become resistant to molecular targeted drugs. Based on these findings and the results of the present study, it is not unreasonable to propose that D40 siRNA is a potential anti-cancer agent.

## Materials and Methods

### Cell Lines

The human cervical cancer cell line HeLa and the human mammary cancer cell line MCF-7 were cultured in Dulbecco’s modified Eagle’s medium (D-MEM,) supplemented with 10% fetal bovine serum (FBS; GIBCO). The human prostate cancer cell line PC-3M-luc-C6 (abbreviated as PC-3M-luc; Xenogen, Alameda, CA) and human mammary cancer cell line MDA-MB231-luc-D3H2LN (abbreviated as MDA-MB231-luc, Xenogen) were cultured with D-MEM/Ham’s F12 supplemented with 10% FBS and 200 μg/ml of Zeocin (Invitrogen, Carlsbad, CA, USA). The cultures were maintained *in vitro* at 37 °C in a humidified atmosphere of 5% CO^2^.

### siRNA and *in vitro* transfection

The synthesized double-stranded short inhibitory RNA against D40 mRNA (D40 siRNA) and control siRNA, which are mixtures of three different sequences (siTRIO), were purchased from B-Bridge International, Inc (Logue Avenue Mountain View, CA, USA). The D40 siRNA sequences are as follows; #1 sense, 5′-ggu aaa agu ccc aua gaa aTT-3′, and antisense, 5′-uuu cua ugg gac uuu uac cTT-3′; #2 sense, 5′-gga cga aag ugu aca gaa aTT-3′, and antisense, 5′-uuu cug uac acu uuc guc cTT-3′; and #3 sense, 5′-gga aaa aac uug ggu guu uTT-3′, and antisense; 5′-aaa cac cca agu uuu uuc cTT-3′. siRNAs were transfected into cell lines using the lipofection reagents Lipofectamine (Life Techonology) or LipoTrust EX Oligo (Hokkaido, System Sci., Sapporo, Japan).

### Western Blot (WB) Analysis of Protein

WB analyses were performed as described previously^5^ with the following modifications: 30 μg of the cell lysates was loaded per lane on an SDS-PAGE gel, electrophoresed, and blotted onto PVDF membranes. Rabbit anti-D40 serum (1:500 dilution), a mouse monoclonal anti-β-actin antibody (1:2000 to 1:5000 dilution), and a mouse monoclonal anti-p53 antibody (1:1000 dilution: MBL, Nagoya, Japan) were used as the primary antibodies. A horseradish peroxidase (HRP)-conjugated goat anti-rabbit IgG antibody and an HRP-conjugated anti-mouse IgG were used as the second antibodies. Chemiluminescent detection was performed using SuperSignal West or Femto (Thermo Fisher Sci., Rockford, IL, USA) with a chemiluminescent detector LAS 1000, and the data were analyzed using the Image Gauge program (FUJI FILM, Tokyo, Japan).

### Growth and Cell Death assay

The cells are stained with a 0.2% Trypan blue /PBS (-) solution, and the number of viable cells and dead cells was counted using a hemocytometer to determine the growth and death rate curves. Nuclear staining of the cells was performed with 20 μg/ml Hoechst 33342. Cell counting kit-8 (Dojindo Lab., Kumamoto, Japan) was also used to determine the growth curve of the transfected cells.

### Detection of the sub-G1 fraction by Flow cytometry

The sub-G1 fraction of the cells was detected using DNA content analyses by staining with propidium iodide. Samples were loaded onto a flow cytometer (FACSCalibur, Becton Dickinson, NJ, USA). Data analyses were performed with Cell Quest 1.0 software (Becton Dickinson) using histograms of the DNA contents in each fraction of the cell cycle.

### Caspase 3/7 assay and cytochrome c release assay

Caspase 3/7 activity assays in the transfected cells were performed using the fluorescent assay kit Apo-ONE Homogeneous Caspase-3/7 Assay (Promega, WI, USA). Fluorescence was quantitatively detected using a multi-mode microplate reader (Synergy 4; BioTek Instrument Inc., Winooski, VT, USA). The cytochrome c release assay was performed using cytoplasmic fraction of the cell lysate and the ELISA assay Quantikine Human Cytochrome c Immunoassay (R&D Systems, Minneapolis, MN, USA). The cytoplasmic fraction of the cell lysate was prepared as described previously^47^. Cytochrome c activity was determined as the amount of the cytochrome c per milligram of cytoplasmic protein.

### Animal Experiments and *in vivo* administration of the siRNA and atelocollagen complex

Animal studies were approved by the Committee for Ethics in Animal Experimentation of National Cancer Center and were performed according to the Guideline for Animal Experiments, drawn up by the committee; these studies met the ethical standards required by the law and the guidelines on the use of experimental animals in Japan.

Two million PC-3M-luc cells were transplanted into the left cardiac ventricle of eight-week-old BALB/c-nu/nu male mice. Atelocollagen was used as a carrier for the *in vivo* administration of siRNA into mice. The siRNA and atelocollagen complexes were prepared as described previously^27^,^28^. Two hundred microliters of the siRNA and atelocollagen complex (50 μg of siRNA, 0.05% Atelocollagen) was injected into the tail veils of nude mice.

One million MDA-MB231-luc cells were transplanted into the mammary glands of 5-week-old C.B17/lcr-scid (scid/scid) female mice. Two hundred microliters of the siRNA and atelocollagen complex (20 μg of siRNA, 0.5% Atelocollagen) was injected into the tumors on scid mice.

### Extracorporeal Bioluminescent detection of tumor growth in mice

Because PC-3M-luc and MDA-MB231-luc cells constitutively express luciferase, the growth rates of the tumors transplanted into mice were monitored extracorporeally by bioluminescence from the tumors after intraperitoneal injection of 150 mg/kg D-luciferin (Promega, Madison, WI). The bioluminescent signals from the tumors were then quantitatively detected using the IVIS imaging system (Xenogen).

### Statistical analysis

Statistical analysis was performed using Student’s t-test. Data are expressed as the mean ± S.D. or S.E., and differences were considered significant when the p value was less than 0.05.

### Human subjects

Because we did not use patient clinical samples and because the experimental protocols, which used established commercial human cell lines, do not need to be approved by a committee at our Institute, we did not include the ethical statement on human subjects.

## Additional Information

**How to cite this article**: Urata, Y. N. *et al.* Targeted Knockdown of the Kinetochore Protein D40/Knl-1 Inhibits Human Cancer in a p53 Status-Independent manner. *Sci. Rep.* **5**, 13676; doi: 10.1038/srep13676 (2015).

## Supplementary Material

Supplementary Information
