# Supplementary material for: Targeted Knockdown of the Kinetochore Protein D40/Knl-1 Inhibits Human Cancer in a p53 Status-Independent Manner
Source: Sci Rep. 2015 Sep 8;5:13676. doi: 10.1038/srep13676 (PMC4562263; doi:10.1038/srep13676)

Title : Targeted Knockdown of the Kinetochore Protein D40/Knl-1 Inhibits Human Cancer in a p53 Status-Independent Manner.

Author List : Yuri N. Urata, Fumitaka Takeshita, Hiroki Tanaka, Takahiro Ochiya and Masato Takimoto

Legends for Supplementary Figures

Supplementary Figure 1.

Depletion of D40 protein by Each D40 siRNA.

Three different siRNAs against D40, #22-#24, which are components of the D40 siRNA Trio, were transfected into the HeLa cell line, and 3 days after transfection D40 protein expression was examined by western blot analysis as described previously. D40 protein was depleted in the cells transfected with each of D40 siRNA or with the D40 siRNA Trio, but no depletion was observed in the cells treated with control siRNA. The bands for D40 and β-actin are indicated by arrows.

Supplementary Figure 2.

Growth inhibition of the HeLa cell line by Each D40 siRNA.

Three different siRNAs against D40, as described above, were transfected into the HeLa cell line. Microphotographs taken 3 days after transfection showed that each of the three D40 siRNAs significantly inhibited the growth of the HeLa cell line.

Significant growth inhibition was observed in the cells transfected with each D40 siRNA and with the D40 siRNA Trio, whereas no inhibition was observed in the cells treated with control siRNA.

Supplementary Figure 3.

D40 siRNA induced the activation of Caspase 3/7 in the HeLa cells.

The HeLa cell line was transfected with D40 and control siRNAs as described previously. Caspase 3/7 acitivities in the transfected cells were determined 48 hrs after transfection with the Caspase-Glo 3/7 Assay (Promega). Higher Caspase 3/7 acitivity was observed in the cells transfected with D40 siRNA than in the control cells.

Supplementary Figure 4.

D40 siRNA induced the increased Cytochrome c release in the HeLa cells.

The HeLa cell line was transfected with siRNA as described previously. Cytochorme c activities in the transfected cells were determined 60 hrs after D40 siRNA transcfection. Higher cytochorme c activity was observed in the cells transfected with D40 siRNA than in the control cells.

Supplementary Figure 5.

Original blot probed with the anti-p53 antibody of Figure 3a Left.

Cropped regions used in the figure within the main text are indicated inside the rectangles. The bands for p53 are indicated by an arrow.

Supplementary Figure 6.

Original blot probed with the anti-β-actin antibody of Figure 3a Left.

Cropped regions used in the figure within the main text are indicated inside the rectangles. The bands for β-actin are indicated by an arrow.

Supplementary Figure 7.

Original blot probed with anti-D40 antibody of Figure 3a Right.

Cropped region used in the figure within the main text are indicated inside the rectangle. The major bands for D40 protein, with a mw of 300 kDa, are indicated by an arrow. Several minor bands lower than the main band were non-specific.

Supplementary Figure 8.

Original blot probed with the anti-β-actin antibody of Figure 3a Right.

Cropped regions used in the figure within main text are indicated inside the rectangle. The bands for β-actin are indicated by an arrow.

Supplementary Figure 9.

Original blot probed with anti- D40 antibody of Figure 4a.

The cropped region used in the figure within main text is indicated inside the rectangle. The major bands for D40 protein, with a mw of 300 kDa, are indicated by an arrow.

Supplementary Figure 10.

Original blot probed with the anti-β-actin antibody of Figure 4a.

The cropped region used in the figure within main text is indicated inside the rectangle. The bands for β-actin are indicated by an arrow.

Supplementary Figure 11.

The metastatic growth of PC-3M-luc cells in the bone tissues of mice.

Two million of PC-3M-luc cells were injected into the left heart ventricle of C.B17/Icr-scid (scid/scid) mice on day zero. On day 28, the metastasis was evaluated by IVIS imaging and confirmed by subsequent necropsy. Histopathological analysis by HE staining confirmed micrometastasis in the knees of mice with bioluminescence signals (upper left). Bone metastatic lesions are indicated by an asterisk mark (upper right). In the no signal-detected mice, no micrometastasis was observed (lower). Bar = 200 μm.

Suppl.Fig.1

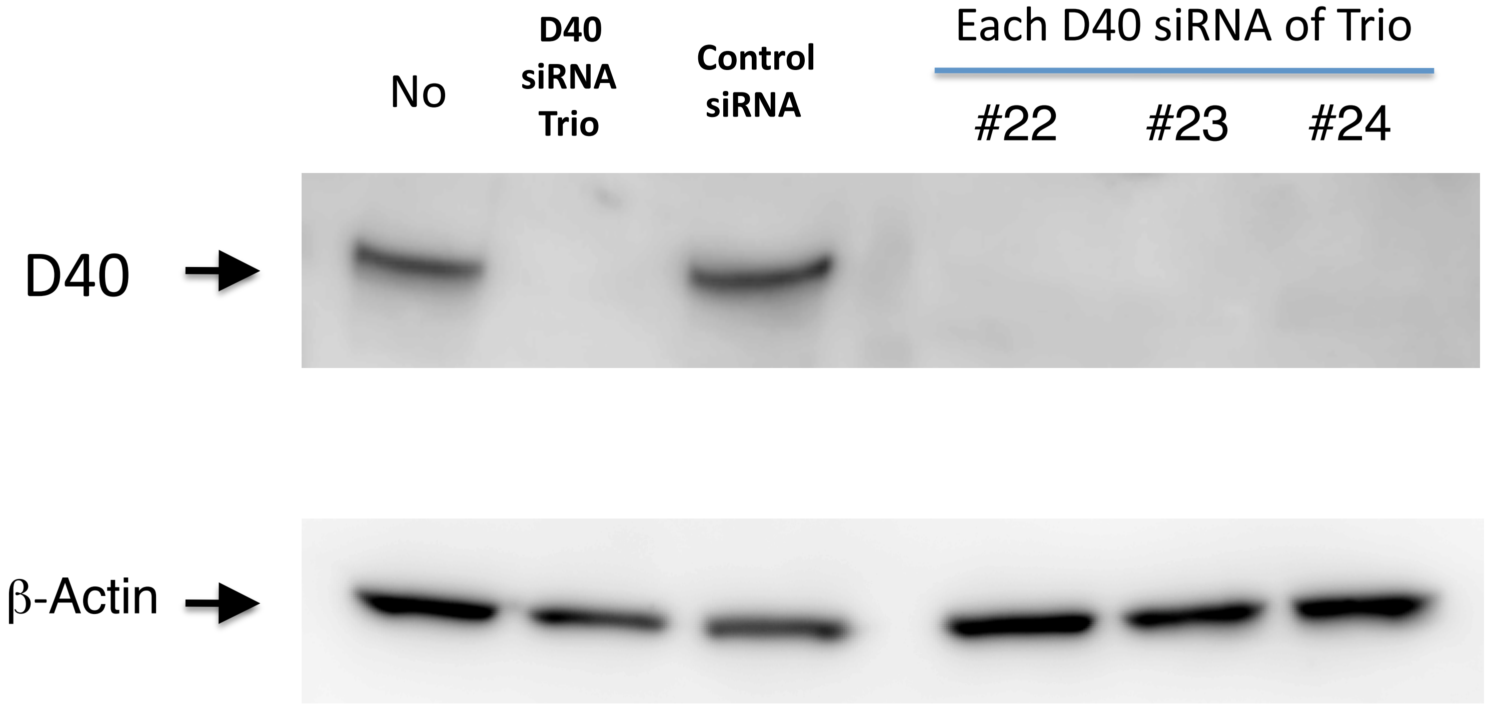
No
D40 siRNA Trio
Control siRNA
Each D40 siRNA of Trio
#22
#23
#24
D40
β-Actin

Suppl.Fig.2

No

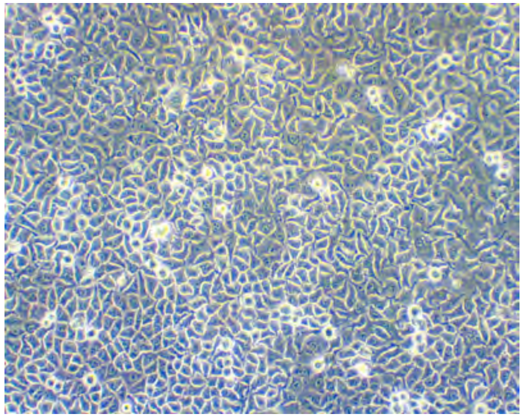

D40 siRNA Trio

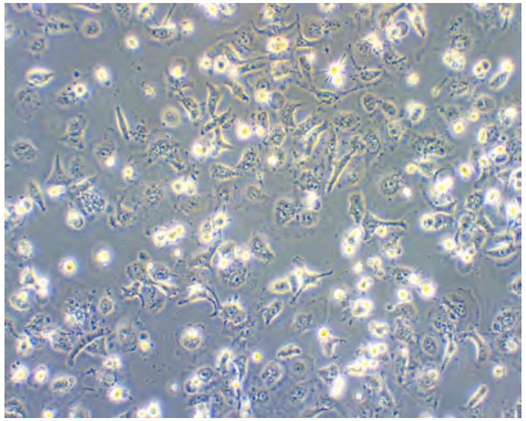

control siRNA

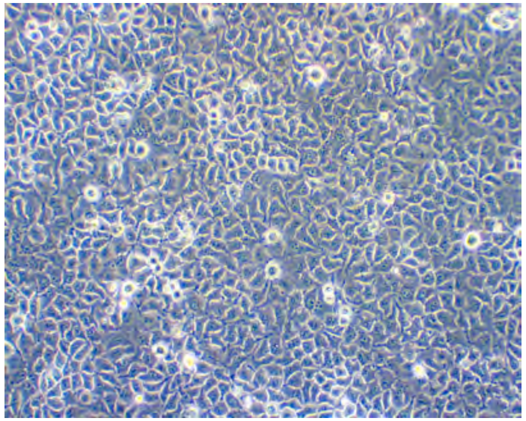

D40 siRNA (#22)

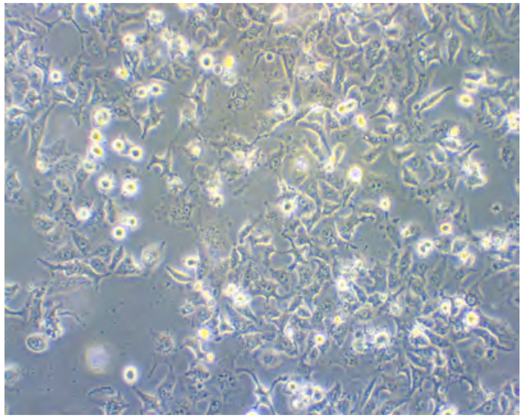

D40 siRNA (#23)

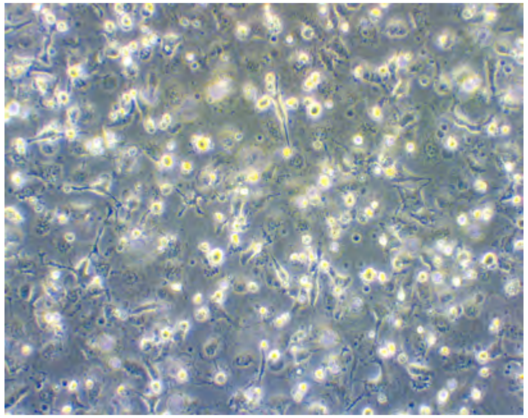

D40 siRNA (#24)

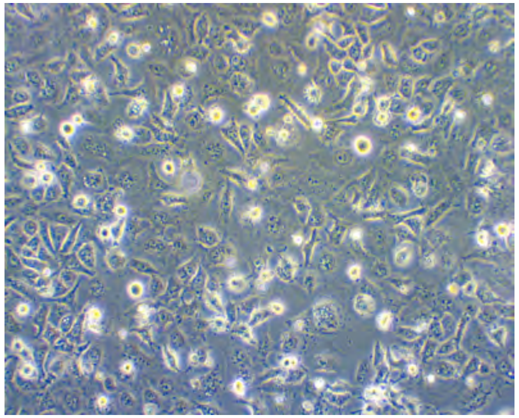

Suppl.Fig.3

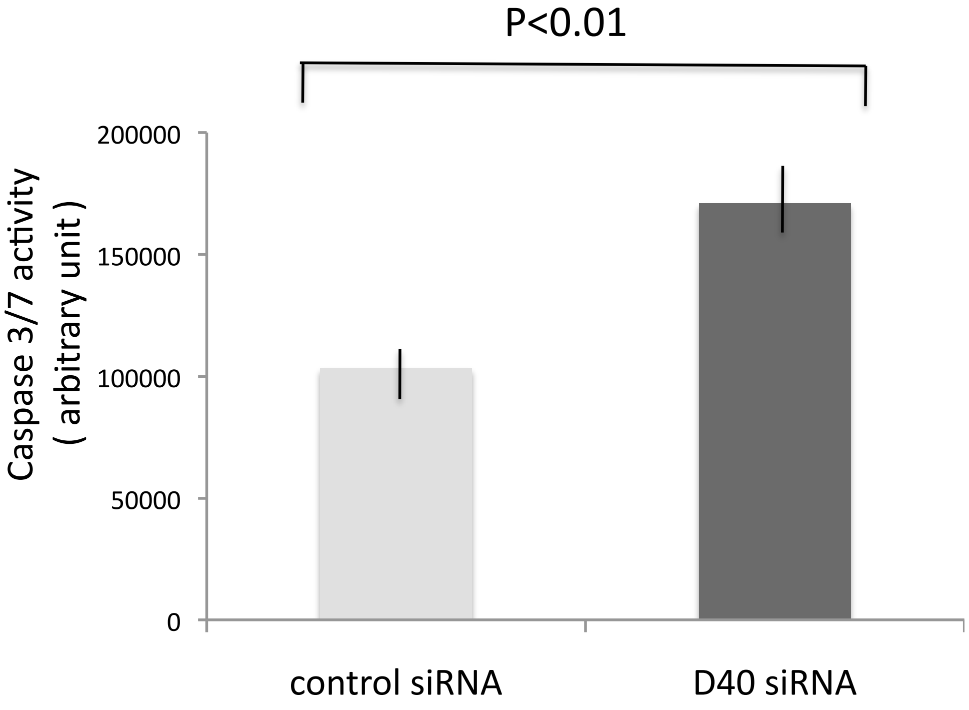

P<0.01
Caspase 3/7 activity
(arbitrary unit)
200000
150000
100000
50000
0
control siRNA
D40 siRNA

Suppl.Fig.4

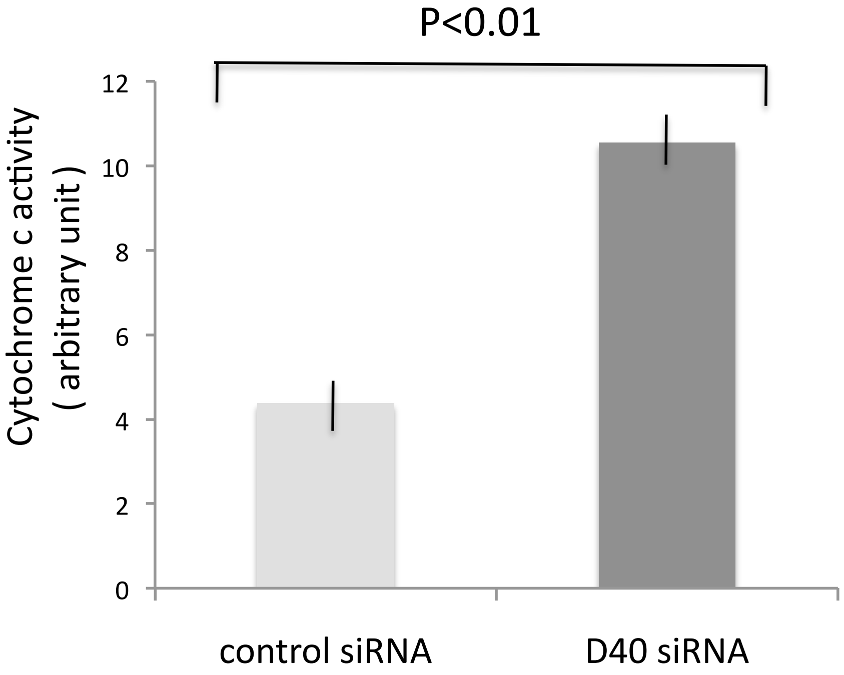

P<0.01
12
10
8
6
4
2
0
Cytochrome c activity
(arbitrary unit)
control siRNA
D40 siRNA

Suppl.Fig.5

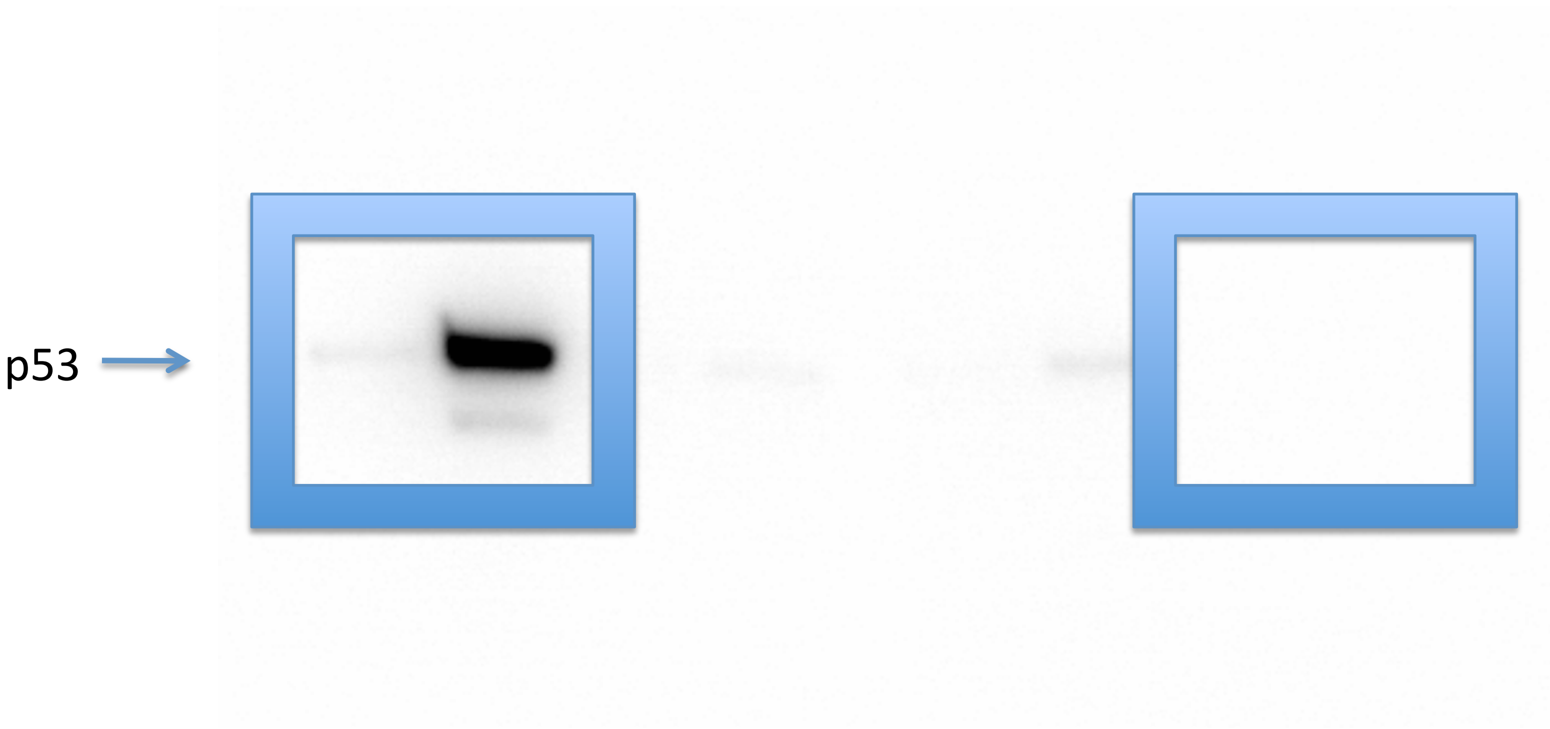

Suppl.Fig.6

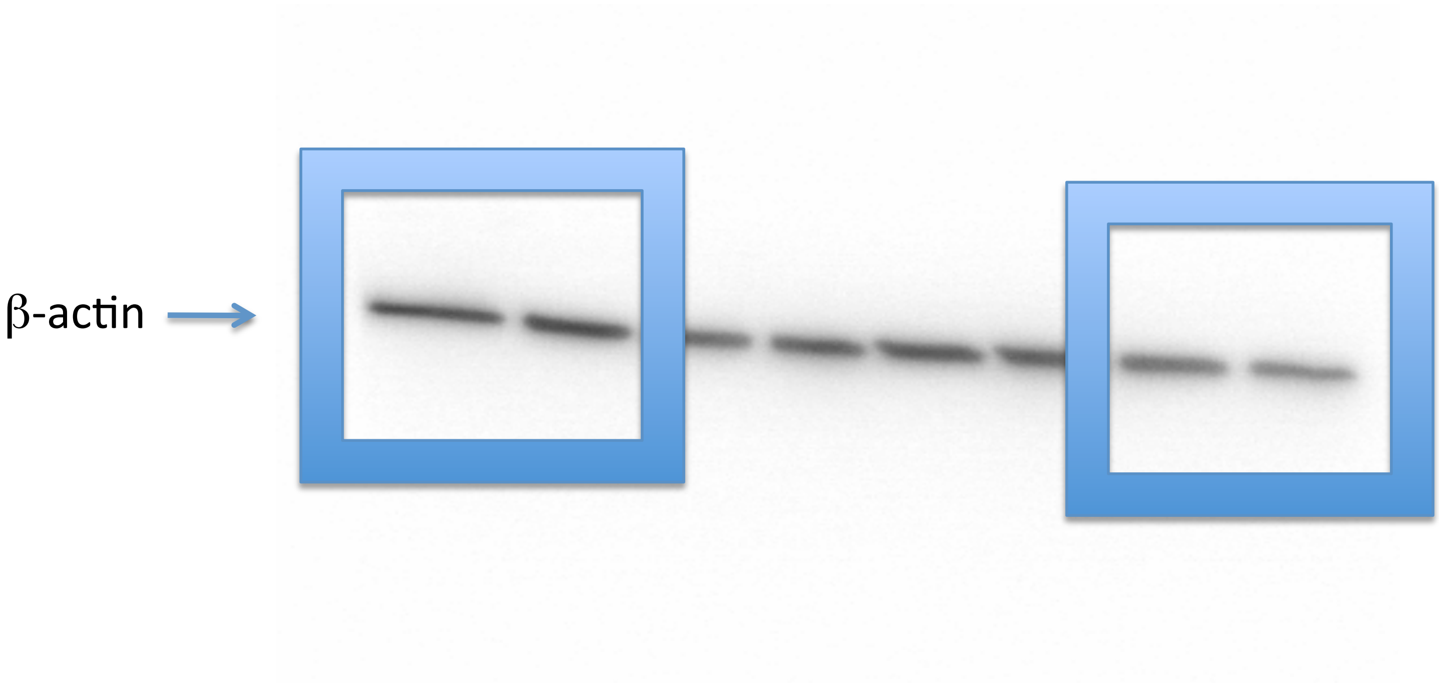

Suppl.Fig.7

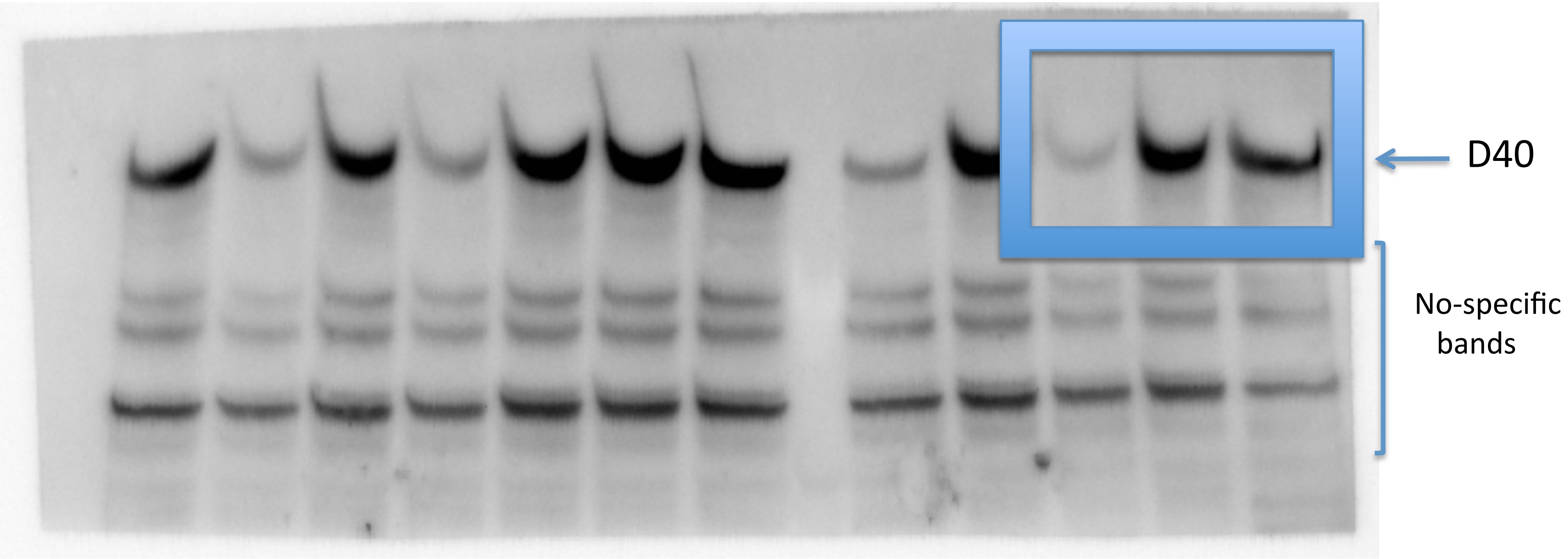

Suppl.Fig.8

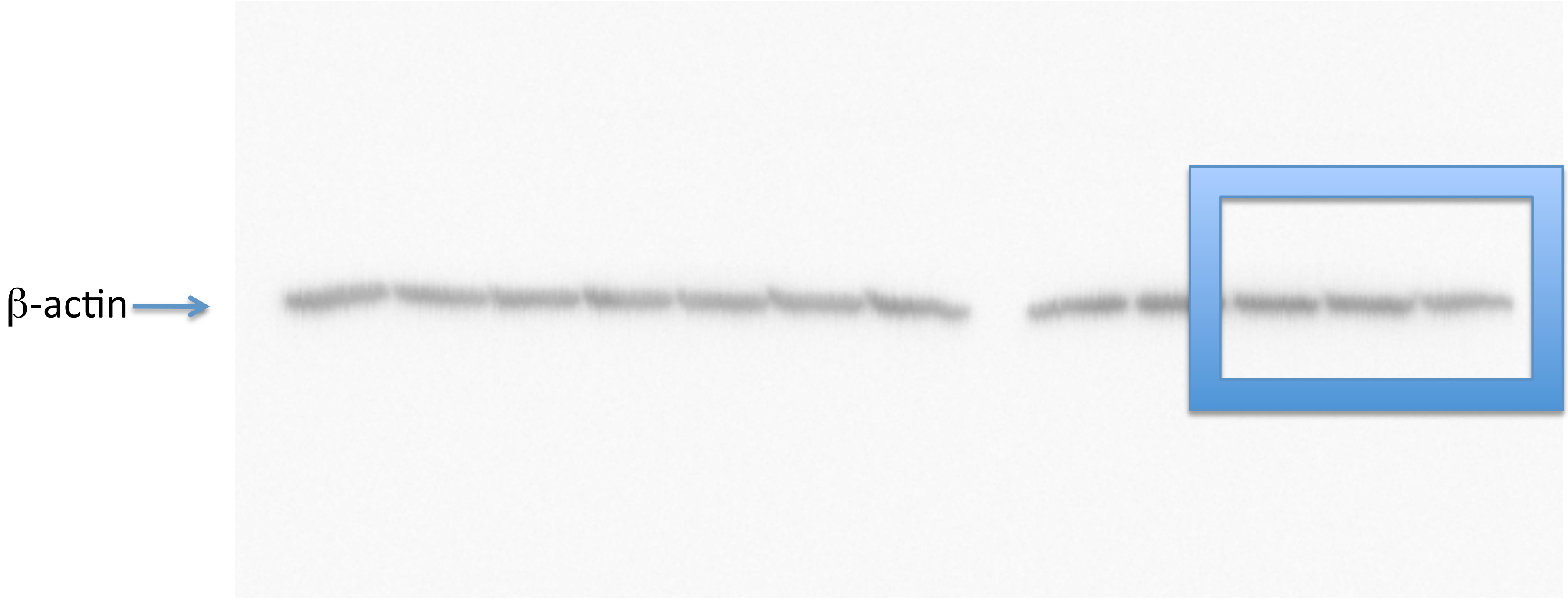

Suppl.Fig.9

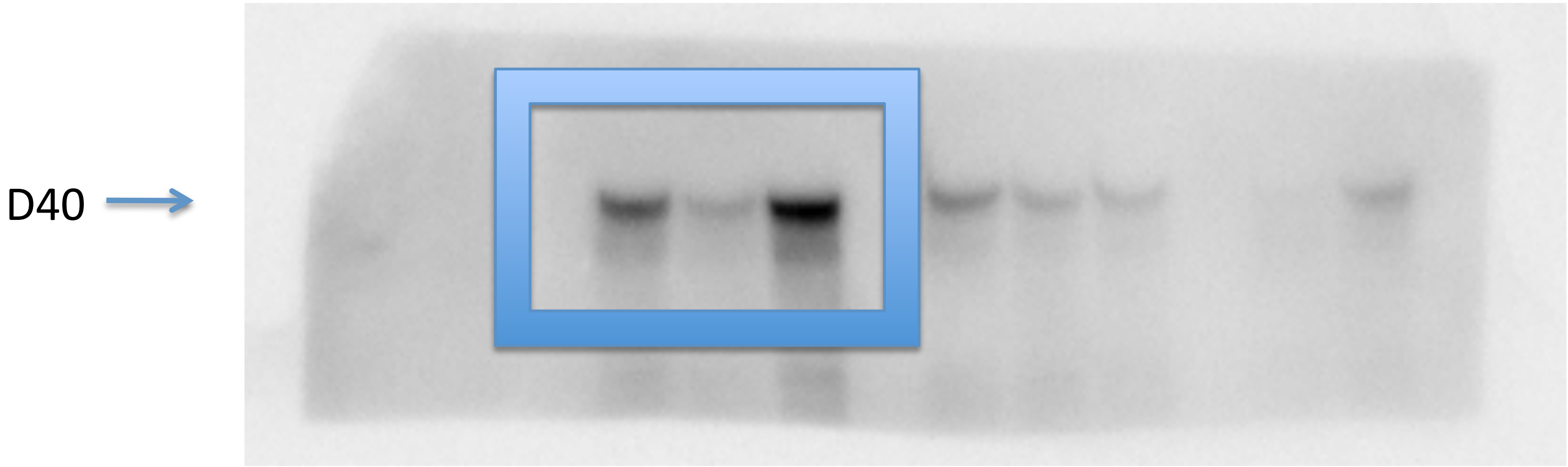

Suppl.Fig.10

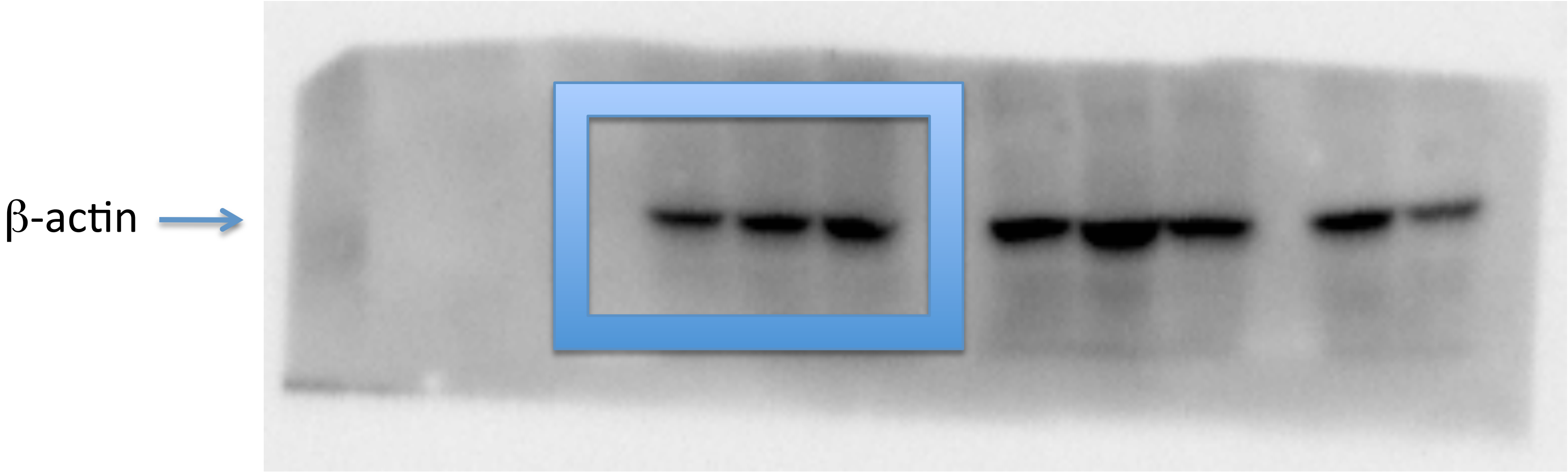

Suppl. Fig.11

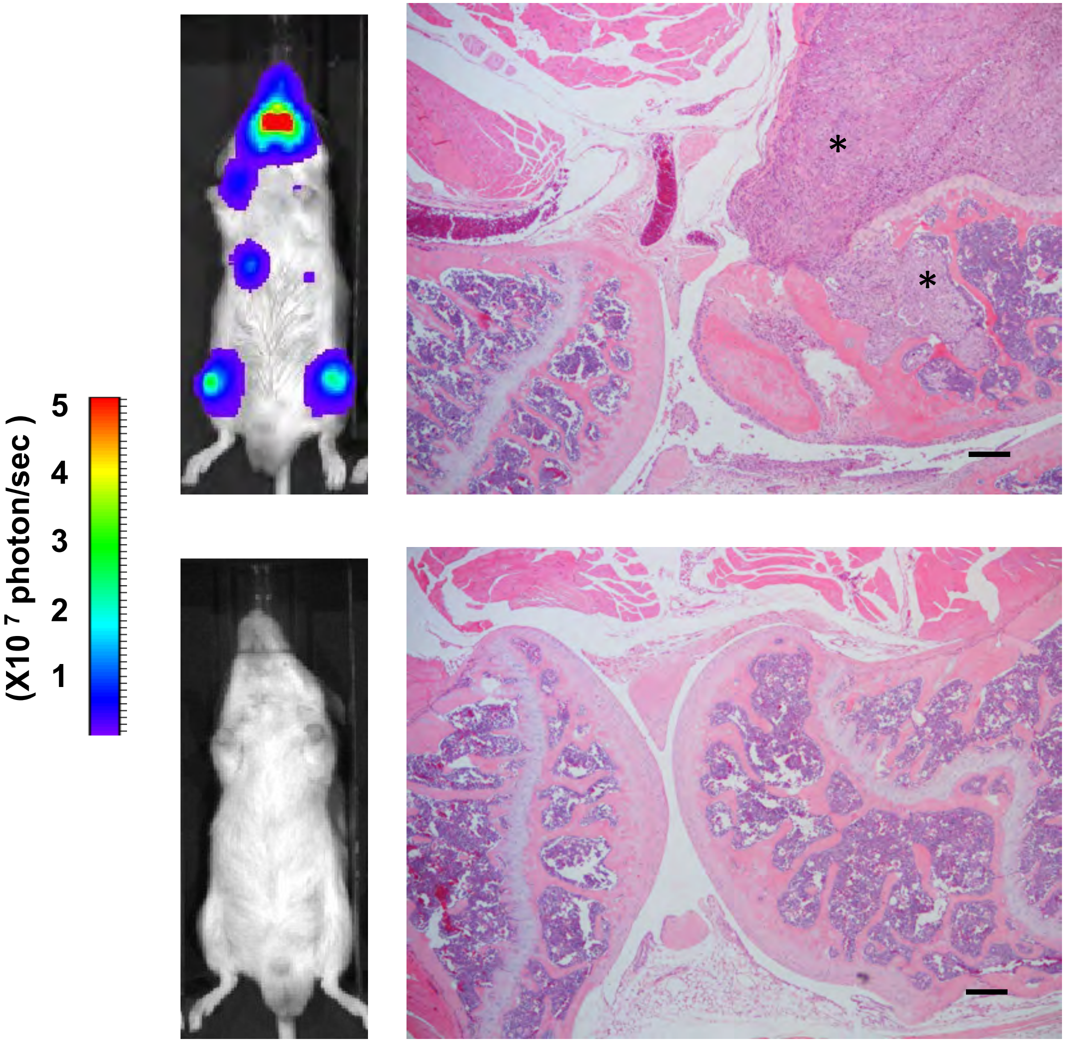

Supplement: Supplementary Information [file srep13676-s1.pdf]
